# Leptin Promotes Greater Ki67 Expression in CD4^+^ T Cells From Obese Compared to Lean Persons Living With HIV

**DOI:** 10.3389/fimmu.2021.796898

**Published:** 2022-01-17

**Authors:** Hubaida Fuseini, Rita Smith, Cindy H. Nochowicz, Joshua D. Simmons, LaToya Hannah, Celestine N. Wanjalla, Curtis L. Gabriel, Mona Mashayekhi, Samuel S. Bailin, Jessica L. Castilho, Alyssa H. Hasty, John R. Koethe, Spyros A. Kalams

**Affiliations:** ^1^ Divison of Infectious Diseases, Vanderbilt University Medical Center, Nashville, TN, United States; ^2^ Tennessee Center for AIDS Research, Vanderbilt University Medical Center, Nashville, TN, United States; ^3^ Division of Diabetes, Endocrinology and Metabolism, Vanderbilt University Medical Center, Nashville, TN, United States; ^4^ Division of Gastroenterology, Hepatology and Nutrition, Vanderbilt University Medical Center, Nashville, TN, United States; ^5^ Department of Molecular Physiology and Biophysics, Vanderbilt University School of Medicine, Nashville, TN, United States; ^6^ The Veterans Affairs Tennessee Healthcare System, Nashville, TN, United States

**Keywords:** leptin, body mass index, obesity, HIV, CD4^+^ T cell, Ki67, IL-17A

## Abstract

While antiretroviral therapy (ART) has proven effective in suppressing viremia and disease progression among people living with human immunodeficiency virus (HIV; PLWH), suboptimal CD4^+^ T cell reconstitution remains a major obstacle in nearly 30% of ART-treated individuals. Epidemiological studies demonstrate that obesity, or a body mass index (BMI) ≥ 30 kg/m^2^, is positively correlated with greater CD4^+^ T cell recovery in PLWH on ART. Leptin is a known immunomodulator that is produced in proportion to fat mass and is increased in obese individuals, including PLWH. We hypothesized that CD4^+^ T cells from obese PLWH have increased cell proliferation and cytokine production compared to cells from lean PLWH, potentially modulated by differential effects of leptin signaling. To test this hypothesis, peripheral blood mononuclear cells from obese and lean PLWH with long-term virologic suppression on the same ART regimen were pretreated with recombinant leptin and then stimulated with anti-CD3/CD28 or PMA/ionomycin to measure Ki67 expression, leptin receptor (LepR) surface expression and cytokine production. In the absence of leptin, Ki67 expression and IL-17A production were significantly higher in CD4^+^ T cells from obese compared to lean PLWH. However, LepR expression was significantly lower on CD4^+^ T cells from obese compared to lean PLWH. After leptin treatment, Ki67 expression was significantly increased in CD4^+^ T cells from obese PLWH compared to the lean participants. Leptin also increased IL-17A production in CD4^+^ T cells from obese healthy controls. In contrast, leptin decreased IL-17A production in CD4^+^ T cells from both obese and lean PLWH. Combined, these results demonstrate that obesity is associated with greater CD4^+^ T cell proliferation among PLWH, and that higher circulating leptin levels in obesity may contribute to improved CD4^+^ T reconstitution in PLWH initiating ART.

## Introduction

CD4^+^ T cells play a critical role in orchestrating immune responses to viral infections, including the human immunodeficiency virus (HIV) (1). HIV infection is associated with progressive loss of CD4^+^ T cells and an impaired immune response to a range of opportunistic infections (2). The reconstitution of circulating CD4^+^ T cells following initiation of antiretroviral therapy (ART) in people living with HIV (PLWH) is an indicator of long-term health outcomes (3–5). While ART exposure effectively suppresses HIV replication to undetectable levels in PLWH, up to 30% of ART-experienced PLWH fail to achieve optimal CD4^+^ T recovery (> 500 cells/mm^3^) (6). Epidemiological studies from the pre-ART era showed that obesity, or a body mass index (BMI) ≥ 30 kg/m^2^, was associated with reduced HIV-associated morbidity and mortality, as well slower disease progression to AIDS (7–9). Now, in the combination ART era, a BMI ≥ 30 kg/m^2^ at treatment initiation is associated with greater CD4^+^ T cell reconstitution (10–12), suggesting that obesity in PLWH initiating ART may potentially play a protective role in immune reconstitution. However, whether obesity can be considered protective remains debatable as higher BMI PLWH have increased systemic inflammation and an increased risk of developing metabolic syndrome (13–19).

Obesity is associated with elevated levels of the adipokine leptin (20), and epidemiological studies demonstrate that PLWH with higher circulating leptin levels have increased CD4^+^ T cell reconstitution compared to those with lower leptin levels (21, 22). Prior studies found that recombinant leptin administration to HIV negative children with congenital leptin deficiency had beneficial effects on neuroendocrine/metabolic dysfunction, as well as on T cell proliferation and cytokine release (23, 24). Based on these initial findings, small clinical trials assessed the effects of recombinant leptin therapy on CD4^+^ T cell reconstitution in PLWH initiating ART. The first trial reported that recombinant leptin administration to eight hypoleptinemic men with HIV on ART improved insulin sensitivity and dyslipidemia, but did not improve CD4^+^ T cell counts (25). In a second trial, the FDA-approved synthetic leptin analog Metreleptin was administered to seven hypoleptinemic men with HIV on ART. Metreleptin improved insulin resistance but again did not improve CD4^+^ T cell counts (26). It is possible that leptin therapy did not improve CD4^+^ T cell counts in these studies because the clinical trials were conducted in PLWH predominantly with a BMI of 22-25 kg/m^2^, or because the participants had been on ART for some time. To date, no studies have assessed the role of leptin therapy early on CD4^+^ T cell reconstitution in obese and/or malnourished PLWH.

Immune cells are known to express the leptin receptor, including CD4^+^ T cells, and leptin has been shown to directly regulate innate and adaptive immune responses (27, 28). Studies in HIV negative individuals and mice show that leptin has a role in T cell thymic development, (29) proliferation of naïve CD4^+^ T cells (30), interferon gamma (IFNγ) production from CD4^+^ T helper 1 (Th1) cells (31, 32) and interleukin 17 (IL-17A) production from CD4^+^ T helper 17 (Th17) cells (33). Furthermore, leptin has been shown to increase toll-like receptor 2 (TLR2) expression on human monocytes (34) and increase the cytotoxic capacity of human natural killer (NK) cells (35). In the context of HIV infection, work from the group led by Dr Víctor Sánchez-Margalet has demonstrated that leptin receptor surface expression is increased on peripheral blood mononuclear cells (PBMCs) from PLWH following phytohemagglutinin (PHA) and Concanavalin A (ConA) stimulation (36). Another study from this group further showed that leptin increased the oxidative burst in monocytes from healthy controls, but decreased the oxidative burst in monocytes from PLWH (37). However, the role of leptin signaling on CD4^+^ T cell proliferation and function in the context of HIV infection remains unclear.

Efforts to determine potential mechanisms for the suboptimal immune reconstitution in some PLWH may be furthered by assessing the effects of leptin signaling on CD4^+^ T cell function in the context of HIV infection. In this study, we investigate the molecular basis by which obesity and leptin signaling mediates CD4^+^ T cell expansion and effector responses *in vitro*. Using flow cytometry, we measured cell proliferation of CD4^+^ T cells from obese and lean PLWH *via* Ki67 expression, a protein expressed in the nuclear region of proliferating cells undergoing cell division (38). Obesity and HIV infection are associated with increased secretion of pro-inflammatory cytokines, including IL-17A (39), and higher IL-17A serum levels and increased Th17 cells (40, 41). We found that CD4^+^ T cell Ki67 expression and IL-17A production were greater in obese compared to lean PLWH. Furthermore, leptin increased Ki67 expression in CD4^+^ T cells from obese compared to lean PLWH. Finally, leptin decreased IL-17A production in CD4^+^ T cells from both obese and lean PLWH, but increased IL-17A production in CD4^+^ T cells from HIV negative controls.

## Materials and Methods

### Study Cohort

We enrolled 26 lean (BMI 18.5-24.9 kg/m^2^) and 35 obese (BMI ≥ 30 kg/m^2^) PLWH from the Vanderbilt Comprehensive Care clinic in Nashville, Tennessee (42, 43). Before enrollment, all persons were on an ART regimen consisting of efavirenz, tenofovir, and emtricitabine (the combination pill *Atripla*) for at least 6 months with plasma viral suppression (HIV-1 RNA viral load <50 copies/mL) for at least 12 months. Additional inclusion criteria were a CD4^+^ T cell count >350 cells/µL at enrollment, no treatment with HMG CoA reductase inhibitors in the prior 6 months, and no known history of cardiovascular diseases, diabetes, or rheumatologic disease. We also enrolled an additional 26 obese HIV negative controls (matched to the obese PLWH by BMI, sex, and race).

### Peripheral Blood Mononuclear Cell Isolation and Cryopreservation

Whole blood samples were collected in EDTA tubes and PBMCs were isolated by Ficoll-Paque density gradient centrifugation. PBMCs were cryopreserved in fetal bovine serum (Atlas Biological, Fort Collins, CO) containing 10% dimethyl sulfoxide (DMSO, Sigma-Aldrich, St. Louis, Missouri).

### Cell Culture and Stimulation Protocol

Cryopreserved PBMCs were rapidly thawed, washed twice in pre-warmed RPMI-1640 medium, and rested overnight in complete RPMI-1640 containing 10% FBS, 1% penicillin/streptomycin, 2 mM L-glutamine, 10 mM HEPES and 1 mM sodium pyruvate (Invitrogen, Carlsbad, CA). The next day, rested PBMCS were resuspended in fresh media and 1x10^6^ cells/well was seeded into a 96 well plate pre-coated with anti-CD3 (2 µg/mL; BD Biosciences, Catalog No. 550368). Soluble anti-CD28 (1 µg/mL; Biolegend, Catalog No. 302934) and anti-CD49d (1 µg/mL; Biolegend, Catalog No. 304340) was added for co-stimulation and the cells stimulated for 2 days at 37⁰ C in 5% CO_2_. In select experiments, cells were stimulated with 50 ng/mL of Phorbol-12-myristate 13-acetate (PMA) (Sigma Aldrich, Catalog No. P1585) and 1 µM of Ionomycin (Sigma Aldrich, Catalog No. 19657), and 0.07% Golgi-plug (BD Biosciences, Catalog No. 555029) for 6 hours in fresh complete RPMI media.

### Plasma Cytokine Measurements

Plasma cytokines and markers of inflammation, including leptin, IFNγ, tumor necrosis factor (TNF)-α, interleukins IL-2, IL-12p70, IL-4, IL-5, IL-13, IL-17A, IL-1β, and IL-6 were measured in duplicate using a multiple immunoassay panel (MesoScale, Rockville, MD) as previously described (42).

### Recombinant Leptin Treatment

In select experiments, rested PBMCs were pre-treated with 0, 5, 10, 50, and 100 nM concentrations of recombinant leptin (R and D systems, Catalog No. 398-LP-01M) for 48 hours in serum-free media (Lonza X VIVO 10 with Gentamicin, Catalog No. 04-380Q). Leptin stock concentrations were diluted in Ultrapure MilliQ water (Sigma Aldrich). Following leptin pre-treatment, cells were stimulated with anti-CD3/CD28/CD49d or PMA/Ionomycin.

### Surface and Intracellular Flow Cytometric Analysis

Following leptin treatment and stimulation, PBMCs were stained with Live/Dead Fixable Aqua Kit (Invitrogen, Catalog No. L34957) and surface stained with the following fluorescent antibodies: APC-Cy7 anti-CD3 (clone OKT), PERCPCY5.5 anti-CD4 (clone RPA-T4), BUV 395 anti-CD8 (clone RPA-T8), V 500 anti-CD19 (clone H1B19), V 500 anti-CD14(clone 1G1), ALEXA FLUOR 647 anti- leptin receptor (clone 52263). Cells were then fixed, permeabilized using the Fopx3/transcription factor staining kit (Tonbo Biosciences, Catalog CatTNB-0607-KIT), and intracellularly stained for BV 711 anti-KI67 (clone B6H12). After stimulation with PMA/Ionomycin, cells were intracellular stained for BV 421 anti-IL-2 (clone 5344.111), PE-Dazzle 594 anti-IL-17A (clone BL168), APC anti-TNFα (clone MAb11), PE-Cy7 anti-IFNγ (clone B27), and PE anti-IL-4 (clone MP4-2502), and PE anti-IL-13(clone JES10-5A2). CD3, IL-17A, IFNγ, IL-4, and IL-13 were from Biolegend. All other antibodies were from BD Biosciences. Data was acquired on a 5 laser LSR II cytometer and evaluated using Flowjo software version 10.

### Statistical Analyses

Clinical and demographic characteristics are expressed as median and inter-quartile range (IQR) or percentage. Differences between lean and obese PLWH, or between obese PLWH and obese HIV negative participants, were calculated using Mann-Whitney t-tests. Associations between % LepR expression in CD4^+^ T cells from obese and lean PLWH versus matched plasma leptin levels or BMI were assessed *via* Spearman’s rank correlations. For leptin treatment experiments, differences between untreated and leptin treated samples were calculated using the Wilcoxon matched-pairs signed rank-sum test. Differences between leptin-treated conditions among obese and lean PLWH, or obese PLWH and obese controls, were calculated using Mann-Whitney t-tests. For all analyses, p < 0.05 was considered statistically significant.

### Study Approval

This study was approved by the Vanderbilt University Medical Center Institutional Review Board and all participants provided written informed consent. The investigators carried out studies by guidelines of the United States Department of Health and Human Services. This study is registered on clinicaltrials.gov (NCT04439448).

## Results

### Demographic and Clinical Characteristics of Study Participants

Obese (*n* = 35) and lean (*n* = 26) PLWH, as well as obese HIV negative controls (*n* = 26), demographic and clinical characteristics are compared in **Table 1**. The median age was similar between obese and lean PLWH (p < 0.077) but significantly higher in obese PLWH compared to obese HIV negative controls (p < 0.004). BMI and plasma leptin levels were statistically different between obese and lean PLWH (p < 0.0001 for both). All other demographic and/or clinical characteristics were similar between obese and lean PLWH, or obese PLWH compared to obese HIV negative controls.

**Table 1:** Demographic and Clinical Characteristics of Study Participants.

Variable	(A) Lean HIV (+) BMI = 18.5 - 24.9 kg/m^2^ n = 26	(B) Obese HIV (+) BMI ≥ 30 kg/m^2^ n = 35	(C) Obese HIV (-) BMI ≥ 30 kg/m^2^ n = 26	*p* – value A vs. B	*p* – value B vs. C
Age, median yr (IQR)	39 (34, 48)	46 (39, 51)	37 (28, 44)	0.077	**0.004**
BMI (kg/m^2^)	22.30 (20.97, 23.81)	35.64 (32.99, 48.97)	36.80 (32.57, 56.60)	**<0.0001**	0.811
Female (%)	5 (19%)	16 (46%)	14 (54%)		
Caucasian (%)	11 (42%)	20 (57%)	8 (31%)		
**IMMUNE MARKERS**					
CD4+ count (cells/µL) at enrollment	589 (504, 822)	758 (605, 966)		0.069	
CD8+ count (cells/µL) at enrollment	642 (542, 869)	796 (627, 1061)		0.193	
CD3+ count (cells/µL) at enrollment	1319 (1059, 1683)	1693 (1408, 1878)		**0.049**	
CD4:CD8 ratio at enrollment	0.970 (0.710, 1.150)	0.980 (0.770, 1.300)		0.562	
**GLUCOSE METABOLISM MEASUREMENTS**					
Hemaglobin A1c	5.3 (4.3, 5.5)	5.2 (4.2, 5.6)	5.2 (4.6, 5.6)	0.496	0.908
Fasting glucose, mg/dL	82(64, 87)	88 (59, 94)	89 (77, 95)	0.347	0.194
**PLASMA LIPIDS**					
Leptin, ng/mL	4.26 (0.97, 7.56)	30.37 (18.33, 42.58)	34.90 (23.08, 47.11)	**<0.0001**	0.173
Cholesterol, mg/dL	172 (111, 184)	177 (116, 200)	157 (107, 182)	0.316	0.192
Fasting LDL, mg/dL	99 (43, 119)	111 (61, 129)	103 (66, 121)	0.130	0.432
Fasting HDL, mg/dL	47 (28, 60)	44 (21, 49)	39 (29, 44)	0.550	0.080
Fasting TG, mg/dL	87 (52, 105)	104 (41, 152)	100 (38, 143)	0.051	0.247
**ART HISTORY/OTHER**					
Duration to ART therapy, yr	5.77 (2.66, 9.09)	16.7 (1.60, 11.2)		0.225	
Smoker (%)	9 (35%)	13 (37%)	4 (15%)		
Hepatitis C (%)	2 (7%)	4 (11%)	0 (0%)		

All values median with interquartile range (IQR). ART, antiretroviral therapy; BMI, body mass index; HDL, high-density lipoproteins; LDL, low-density lipoproteins; TG, triglycerides; yr, year. The bolded are p < 0.05, indicating statistical significance.

### Ki67 Expression Is Increased in CD4^+^ T Cells From Obese PLWH Compared to Cells From Lean PLWH

**Figure 1:**
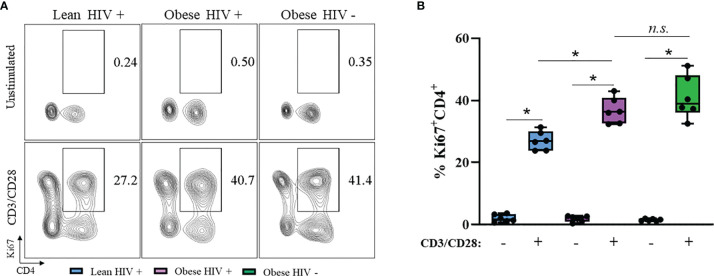
Ki67 expression is increased in CD4^+^ T cells from obese PLWH compared to lean PLWH. PBMCs from obese and lean PLWH, as well as HIV negative obese controls, were stimulated with anti-CD3/CD28/CD49d for 2 days. **(A)** Representative flow gating of Ki67+CD4+ T cells and **(B)** Quantification of the percentage of Ki67^+^CD4^+^ T cells. Mann-Whitney tests were used to compare obese and lean PLWH, or obese PLWH and obese HIV negative controls. n = 5-6 persons per group. *p < 0.05 was considered statistically significant. Data shows one representative experiment repeated three times. *n.s*. means not statistically significant.

A higher BMI (≥ 30 kg/m^2^) at the initiation of ART in PLWH is correlated with greater CD4^+^ T cell recovery (12), thus we hypothesized that CD4^+^ T cells from obese PLWH have increased proliferation compared to CD4^+^ T cells from lean PLWH. To test this hypothesis, PBMCs from obese and lean PLWH, as well as obese HIV negative controls, were stimulated with anti-CD3/CD28/CD49d for 2 days. We then measured intracellular expression of the cell proliferation marker Ki67. Compared to unstimulated conditions, Ki67 expression was significantly increased with anti-CD3 stimulation in all three groups (**Figure 1A**). The percentage of Ki67 expressing CD4^+^ T cells was significantly increased in obese PLWH compared to cells from lean counterparts (**Figure 1B**). There was no significant difference in Ki67 expression in CD4^+^ T cells from obese PLWH compared to obese HIV negative controls (**Figure 1B**). We also measured Ki67 expression in CD8^+^ T cells following anti-CD3 stimulation and found no differences in Ki67 expression between obese and lean PLWH (**Supplemental Figure 1B**). However, Ki67 expression was significantly increased in CD8^+^ T cells from obese PLWH compared to obese HIV negative controls (**Supplemental Figure 1B**).

### IL-17A Production Is Increased in CD4^+^ T Cells From Obese PLWH Compared to Cells From Lean PLWH

**Figure 2:**
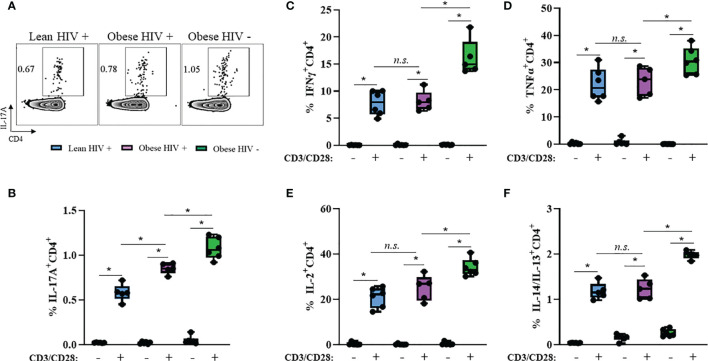
IL-17A production is increased in CD4^+^ T cells from obese PLWH compared to lean PLWH. PBMCs from obese and lean PLWH, as well as obese HIV negative controls, were stimulated with PMA/Ionomycin for 6 hours in the presence of 0.07% Golgi-Plug, and intracellular cytokine production was measured by flow cytometry. **(A)** Representative flow gating of IL-17A expression in CD4+ T cells following stimulation. Quantification of the percentage of CD4+ T cells producing **(B)** IL-17A, **(C)** IFNγ, **(D)** TNFα **(E)**, IL-2 **(F)** and IL-4/IL-13. Mann-Whitney tests were used to compare obese and lean PLWH, or obese PLWH and obese HIV negative controls. n = 5-6 persons per group. *p < 0.05 was considered statistically significant. Data shows one representative experiment repeated three times. *n.s*. means not statistically significant.

Obesity and HIV infection are associated with increased local and systemic inflammation, mediated by several cytokines including IFNγ, TNFα, IL-2, IL-4, IL-13, and IL-17 (42). We measured plasma levels of these cytokines between groups and found no statistical differences in expression levels (**Supplemental Figure 2**). Long-term ART exposure is associated with decreased expression of these pro-inflammatory cytokines (39) and may have influenced why we did not observe differences. Therefore, we decided to assess the cytokine production capacity of cells *in vitro via* intracellular flow cytometry. PBMCs from obese and lean PLWH, as well obese HIV negative controls, were stimulated with PMA/Ionomycin in the presence of a protein-transport inhibitor to measure intracellular cytokine production. PMA/Ionomycin stimulation significantly increased cytokine production in CD4^+^ T cells (**Figures 2A–F**). IL-17A production was significantly increased in CD4^+^ T cells from obese PLWH compared to lean PLWH (**Figures 2A, B**). However, we found no significant differences in IFNγ, TNFα, IL-2, IL-4 and IL-13 production between CD4^+^ T cells from obese and lean PLWH (**Figures 2C–F**). CD4^+^ T cells from obese HIV negative controls had increased IFNγ, TNFα, IL-2, IL-4, IL-13 and L-17A production compared to CD4^+^ T cells from obese PLWH (**Figures 2B–F**).

### Leptin Receptor Expression Is Decreased on CD4^+^ T Cells From Obese PLWH Compared to Cells From Lean PLWH

**Figure 3:**
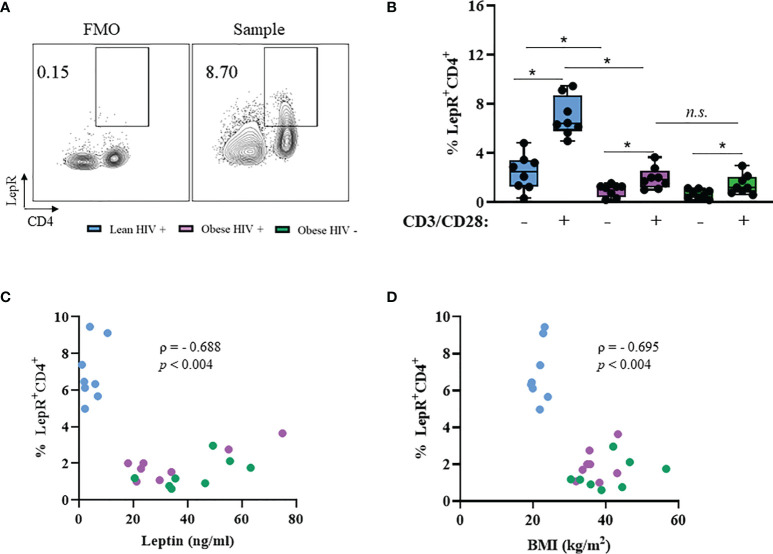
Leptin receptor expression is decreased in CD4^+^ T cells from obese PLWH compared to lean PLWH. PBMCs from obese and lean PLWH, as well obese HIV negative controls, were stimulated with anti-CD3/CD28/CD49d for 2 days. **(A)** Representative flow gating of leptin receptor (LepR) surface expression in CD4+ T cells following activation. **(B)** Quantification of the percentage of LepR+CD4+ T cells. Relationship between percentage of LepR+CD4+ cells and **(C)** plasma leptin levels or **(D)** by BMI. In **(B)**, Mann-Whitney tests were used to compare obese and lean PLWH, or obese PLWH and obese HIV negative controls. n = 8-9 persons per group. *p < 0.05 was considered statistically significant. Data shows one representative experiment repeated three times. *n.s*. means not statistically significant. In **(C, D)**, Spearman’s rank correlations were conducted for plasma leptin and BMI measurements.

PBMCs from PLWH are known to express the leptin receptor (LepR) (36), However, it is unclear whether LepR is differentially expressed on CD4^+^ T cells from obese and lean PLWH. PBMCs from obese and lean PLWH, and obese HIV negative controls, were stimulated with anti-CD3/CD28/CD49df for 2 days and LepR surface expression was measured *via* flow cytometry. Compared to unstimulated conditions, anti-CD3 stimulation increased LepR expression on CD4^+^ T cells in all three groups (**Figures 3A, B**). LepR expression was significantly lower on CD4^+^ T cells from obese PLWH compared to lean PLWH (**Figure 3B**). However, we found no significant difference in LepR expression between CD4^+^ T cells from obese PLWH and obese HIV negative controls. We next performed Spearman’s rank correlations to determine if there was a relationship between LepR expression on CD4^+^ T cells and plasma leptin levels or BMI. LepR expression was negatively correlated with plasma leptin levels r= -0.688, p=0.004 (**Figure 3C**) and BMI status (r= -0.695, p= 0.004) (**Figure 3D**).

### Leptin Increased CD4^+^ T Cell Proliferation in Obese PLWH Compared to Cells From Lean PLWH

**Figure 4:**
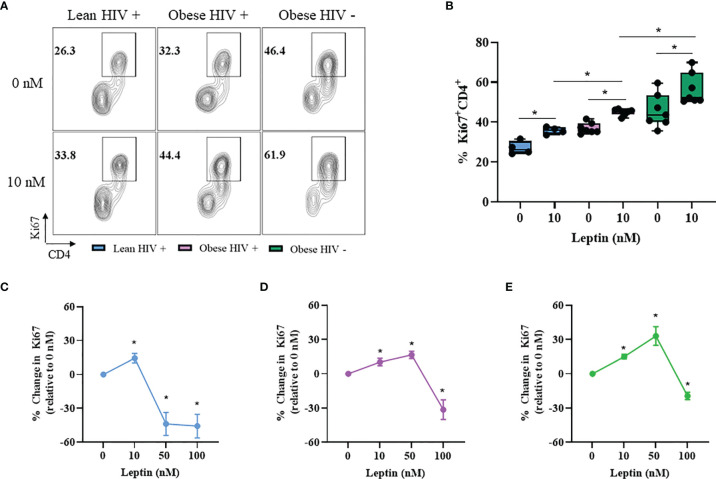
Leptin increased CD4^+^ T cell proliferation in obese compared to lean PLWH. PBMCs from obese and lean PLWH, as well as obese HIV negative controls, were pre-treated with recombinant leptin at the indicated concentrations for 48 hours, then stimulated with anti-CD3/CD28/CD49d for 2 days. **(A)** Representative flow gating of Ki67^+^CD4^+^ T cells in obese and lean PLWH and HIV negative controls after 0 or 10 nM leptin treatment. **(B)** Quantification of the percentage of Ki67^+^CD4^+^ T cells under 0 or 10 nM leptin treatment. Quantification of the percentage change in Ki67 expression (relative to 0 nM conditions) in CD4+ T cells from **(C)** lean PLWH, **(D)** obese PLWH and **(E)** obese HIV negative control following 0, 10, 50 or 100 nM leptin treatment. In **(B)**, Wilcoxon-matched pairs rank signed tests were used to compare 0 and 10 nM values for each person. Mann-Whitney tests were used to compare 10 nM leptin treatment between groups. In **(C–E)**, Kruskal-Wallis tests were used to compare the relative change compared to vehicle treatment in each metabolic group. n = 4-7 people per group. *p < 0.05 was considered statistically significant. Data shows one representative experiment repeated two times.

Leptin has immunomodulatory functions (28) and studies by Sánchez-Margalet et al. previously demonstrated that the maximal effect of leptin on CD4^+^ T cell proliferation and activation was observed with 10 nM leptin treatment (30). However, it is unclear whether leptin signaling differentially modulates CD4^+^ T cell proliferation in obese and lean PLWH. To determine the effect of leptin on CD4^+^ T cell proliferation, we pre-treated PBMCs from obese and lean PLWH, as well as obese HIV negative controls, with 10 nM leptin for 48 hours in serum free media. The cells were then stimulated with anti-CD3/CD28/CD49d for 2 days and Ki67 expression measured by flow cytometry. Leptin significantly increased Ki67 expression in CD4^+^ T cells from obese and lean PLWH (**Figure 4A**). Furthermore, leptin-induced Ki67 expression was greater in CD4^+^ T cells from obese PLWH compared to lean PLWH. Additionally, leptin-induced Ki67 expression was significantly increased in CD4^+^ T cells from obese HIV negative controls compared to CD4^+^ T cells from obese PLWH (**Figure 4B**).

CD4^+^ T cells are reported to retain immunological memory of obesity status (44), thus we hypothesized that since CD4^+^ T cells from obese PLWH are exposed to higher circulating leptin levels *in vivo*, it is likely that immunological memory of this high leptin abundant environment may differently effect CD4^+^ T cell activation and effector responses following *ex vivo* stimulation. To test this hypothesis, we pre-treated CD4^+^ T cells from obese and lean PLWH with leptin concentrations known to elicit responses in T cells (0, 10, 50 and 100 nM) (35) and measured the change in Ki67 expression relative to 0 nM leptin treatment conditions (**Figures 4C–E**). Similar to our findings in **Figure 4B**, 10 nM leptin treatment significantly increased Ki67 expression in CD4^+^ T cells in obese and lean PLWH. However, 50 nM leptin treatment further augmented Ki67 expression in CD4^+^ T cells from obese PLWH, but significantly deceased Ki67 expression in cells from lean PLWH (**Figures 4C, D**). 50 nM leptin treatment also augmented Ki67 expression in CD4^+^ T cells from obese HIV negative controls (**Figure 4E**). With 100 nM of leptin treatment, Ki67 expression is significantly decreased in CD4^+^ T cells from both obese and lean PLWH, and obese HIV negative controls (**Figures 4C–E**).

### Leptin Decreased IL-17A Production in CD4^+^ T Cells From Obese and Lean PLWH, But Increased IL-17A Production in CD4^+^ T Cells From Obese HIV Negative Controls

**Figure 5:**
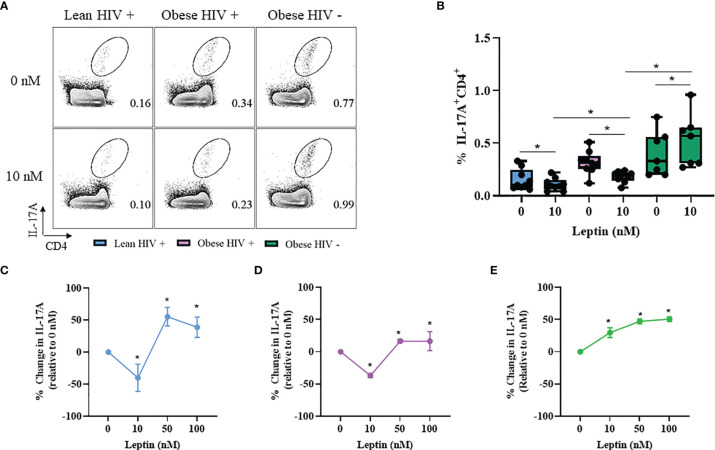
Leptin decreased IL-17A production in CD4^+^ T cells from obese and lean PLWH but increased IL-17A production in CD4^+^ T cells from obese HIV-negative controls. PBMCs from obese and lean PLWH as well as obese HIV negative controls were pre-treated with 0 stimulated with PMA/Ionomycin for 6 hours in the presence of 0.07% Golgi-Plug. **(A)** Representative flow gating of IL-17A+CD4+ T cells following 0 or 10 nM leptin treatment conditions. **(B)** Quantification of the percentage of IL-17A+CD4+ T cells following 0 or 10 nM leptin treatment conditions. Quantification of the percentage change in IL-17A production (relative to 0 nM conditions) in CD4+ T cells from **(C)** lean PLWH, **(D)** obese PLWH and **(E)** Obese HIV negative control following 0, 10, 50 or 100 nM leptin treatment. In **(B)**, Wilcoxon-matched pairs rank signed tests were used to compare 0 and 10 nM values for each person. Mann-Whitney tests were used to compare 10 nM leptin treatment between metabolic groups. In **(C–E)**, Kruskal-Wallis were used to compare the relative change compared to vehicle treatment in each metabolic group. n = 4-7 people per group. *p < 0.05 was considered statistically significant. Data shows one representative experiment repeated two times.

Our results from **Figure 2** showed that IL-17A production was significantly higher in CD4^+^ T cells from obese PLWH compared to lean PLWH; however it remained unclear whether leptin differentially modulates IL-17A production in CD4^+^ T cells from obese and lean PLWH. PBMCs from obese and lean PLWH, as well as HIV negative controls, were pre-treated with 10 nM of recombinant leptin and then stimulated with PMA/Ionomycin for 6 hours in the presence of a protein-transport inhibitor to measure intracellular IL-17A production by flow cytometry. Leptin treatment (10 nM) significantly decreased IL-17A production in CD4^+^ T cells from both obese and lean PLWH, but increased IL-17A production in CD4^+^ T cells from HIV negative controls (**Figures 5A, B**). To determine if the concept of immunological memory referenced in **Figure 4** would apply to IL-17A cytokine secretion, we measured intracellular IL-17A production in CD4^+^ T cells under varying leptin concentrations (0, 10, 50 and 100 nM). The change in IL-17A production, relative to 0 nM culture conditions, was significantly decreased in CD4^+^ T cells from both obese and lean PLWH following 10 nM leptin treatment (**Figures 5C, D**). However, 50 nM and 100 nM leptin treatment significantly increased IL-17A production in CD4^+^ T cells from obese and lean PLWH (**Figures 5C, D**). Furthermore, the relative change in IL-17A production was significantly increased in CD4^+^ T cells from obese HIV negative controls after 10, 50 and 100 nM leptin treatment (**Figure 5E**).

## Discussion

We found that stimulation increased Ki67 expression in CD4^+^ T cells from obese PLWH compared to lean PLWH, suggesting that obesity may promote increased CD4^+^ T cell proliferation in the context of HIV infection. These findings are consistent with the published literature in HIV negative persons, where obesity was associated with increased CD4^+^ T cell numbers (45). We also found that IL-17A production was increased in CD4^+^ T cells from obese PLWH compared to lean PLWH. IL-17A is known to play a critical role in obesity and HIV infection (40, 41), and inhibition of the IL-17A signaling pathway in adipocytes has been shown to attenuate diet-induced obesity in mice (46). Thus, our findings in **Figure 3** contribute valuable information on the *in vitro* effects of obesity on CD4^+^ T cell effector responses in PLWH on long-term ART.

In the absence of leptin treatment, we found that leptin receptor (LepR) surface expression was significantly decreased on CD4^+^ T cells from obese PLWH compared to cells from lean counterparts. These results expand on prior studies in healthy humans, where obesity was correlated with decreased levels of circulating soluble leptin receptor (47). Circulating leptin levels are increased in obesity and, with prolonged exposure, leptin can induce its own negative feedback loop *via* STAT3-dependent SOCS3 accumulation (48–50). It is likely that the combination of HIV infection and long exposure to higher circulating leptin levels in CD4^+^ T cells from obese PLWH may contribute to a negative feedback loop, leading to decreased LepR expression. Our group has recently published that leptin levels mediate the association between body composition, glucose tolerance and systemic inflammation in PLWH initiating ART (51), suggesting that leptin may serve as an independent factor in immune function that is related to, but not colinear with, fat mass. This is supported by our results demonstrating that leptin receptor surface expression was decreased on CD4^+^ T cells from obese PLWH compared to cells from lean PLWH. Future studies should assess leptin receptor gene signatures and leptin secretion as potential indicators of metabolic and immunologic health in PLWH.

CD4^+^ T cells are known to mediate immunological memory of obesity status (44, 52), and since CD4^+^ T cells from obese PLWH are exposed to higher circulating leptin levels *in vivo*, it is possible that an immunological memory of this high leptin environment may be imprinted on CD4^+^ T cells and would potentially impact responses following restimulation. Here, we report that 50 nM leptin treatment augmented Ki67 expression in CD4^+^ T cells from obese PLWH, but CD4^+^ T cells from lean counterparts treated with the same leptin concentration had significantly lower Ki67 expression. The difference in response to leptin treatment at the 10 nM and 50 nM concentrations could reflect immunological memory of prior exposure to higher leptin levels *in vivo*, and may account for the greater responsiveness of CD4^+^ T cells from obese PLWH relative to cells from lean PLWH.

Leptin treatment (10 nM) significantly decreased IL-17A production in CD4^+^ T cells from both obese and lean PLWH, but increased IL-17A production in cells from obese HIV negative subjects. These findings are consistent with a prior study where 10 nM leptin treatment decreased the oxidative burst in monocytes from PLWH, but increased the oxidative burst in monocytes from healthy negative controls (37). HIV-infection is associated with chronic T cell activation and exhaustion (53), thus is likely that leptin’s ability to augment cytokine secretion in exhausted CD4^+^ T cells may be impaired in PLWH. Our findings also suggest that the effect of leptin on IL-17A production in CD4^+^ T cells is not influenced by obesity status. Since it is known that leptin can modulate immune cell effector responses independent of body composition, it is possible that leptin may be modulating IL-17A production in CD4^+^ T cells independent of fat mass.

To determine whether a higher leptin concentration was sufficient to augment IL-17A production in CD4^+^ T cells from the HIV-infected individuals, we treated the cells with 50 nM of leptin. Higher leptin concentrations increased IL-17A production in CD4^+^ T cells from obese and lean PLWH. HIV infection and ART exposure are known to modulate adipocyte function and leptin secretion (54), thus it likely that these two factors may alter the leptin sensitivity of CD4^+^ T cells such that higher leptin concentrations are required to augment cytokine production. The IL-17A signaling axis is also implicated in gastrointestinal inflammatory disorders that perturb bacterial diversity (55), and despite effective viral suppression by ART, HIV infection is associated with abnormal changes to the gut immune environment and impaired reconstitution of gut-resident Th17 cells. Future studies should assess the role of leptin on gut-resident CD4^+^ T cell recovery in PLWH.

A limitation of our study was the lack of lean HIV negative controls. The study cohort for this work was originally created to assess the effect of obesity on various immunological endpoints among PLWH, and included an obese HIV negative group to assess if any differences are specific to PLWH. Therefore a lean HIV negative control group was not recruited. It is possible that decreased LepR surface expression on CD4^+^ T cells from obese PLWH compared to cells from lean PLWH may potentially be mediated by a leptin signaling negative feedback loop involving a STAT3-dependent SOC3 accumulation (48, 50). Unfortunately, we did not have PBMCs remaining from our lean PLWH to conduct western blot analysis to measure expression levels of STAT 3 (total and phosphorylated) as well SOC3. We plan to explore this mechanism further in future studies. Lastly, non-obese and obese participants were recruited based on BMI criteria, which cannot capture differences in lean versus fat mass, as well as fat partitioning, that may impact leptin levels.

Circulating leptin levels are higher in women compared to men with a similar BMI (52) and CD4^+^ T cells from women and female mice have increased cell proliferation and IL-17A production (56). Thus, it is possible that that a sex-specific bias in leptin signaling may differentially modulate CD4^+^ T cell function in HIV-positive obese men and women. Second, leptin has been shown to augment cytokine production in CD4^+^ T cells from malnourished children (57) and a low BMI at ART initiation is correlated with decreased CD4^+^ T cell recovery in PLWH (22). Future studies should determine the *in vitro* effects of leptin treatment on CD4^+^ T cell function in malnourished (low BMI) PLWH, which may inform the need for future clinical trials focused on assessing the efficacy of recombinant leptin supplementation in specific groups of PLWH initiating ART.

Suboptimal CD4^+^ T cells reconstitution remains a barrier to immune recovery for some ART-treated individuals, despite suppression of plasma viremia (5). Leptin, an adipokine produced in proportion to fat mass, is associated with higher CD4^+^ T cell recovery in PLWH, however its role in regulating CD4^+^ T cell proliferation and effector responses in PLWH remained unclear. In this study, we show that obesity is associated with increased CD4^+^ T cell proliferation and IL-17A production in PLWH. Furthermore, leptin stimulation increased CD4^+^ T cell proliferation in obese PLWH relative to cells from lean counterparts. Our results suggest a mechanistic link between leptin and CD4^+^ T cell reconstitution in PLWH on long-term ART that may serve to open new therapeutic avenues in the future.

## Data Availability Statement

The raw data supporting the conclusions of this article will be made available by the authors, without undue reservation.

## Ethics Statement

The studies involving human participants were reviewed and approved by Vanderbilt University Medical Center Institutional Review Board. The patients/participants provided their written informed consent to participate in this study.

## Author Contributions

HF, JK, and SK: conceptualization and methodology. Resources: RS, CN, JS, LH, JK, and SK. Formal data curation: HF, RS, CN, JS, LH, and JK. Validation: HF, JK, and SK. Investigation: CW, CG, MM, SB, and JC. Supervision: AH, JK, and SK. Analysis and statistics: HF, JK, and SK. Writing original draft: HF, AH, JK, and SK. Funding acquisition: JK and SK. All authors - review and editing. All authors contributed to the article and approved the submitted version.

## Funding

This work was supported by National Institutes of Health grants K23 AI100700 and R01 DK112262, the Vanderbilt Clinical and Translational Science award from NCRR/NIH grant UL1 RR024975, and the Tennessee Center for AIDS Research grant P30 AI110527. The funding authorities had no role in study design; data collection, analysis, or interpretation; decision to publish; or preparation of the manuscript.

## Conflict of Interest

The authors declare that the research was conducted in the absence of any commercial or financial relationships that could be construed as a potential conflict of interest.

## Publisher’s Note

All claims expressed in this article are solely those of the authors and do not necessarily represent those of their affiliated organizations, or those of the publisher, the editors and the reviewers. Any product that may be evaluated in this article, or claim that may be made by its manufacturer, is not guaranteed or endorsed by the publisher.

## Supplementary Material

Supplementary Figure 1 Gating Strategy. **(A)** Gating Scheme, following selection of singlet viable CD3+CD19-CD14- T cells, we identified Ki67+CD4+ T cells, leptin receptor (LepR)+CD4+ T cells or IL-17A+CD4+T cells. **(B)** Quantification of the percentage of Ki67+CD8+T cells after anti-CD3/CD28/CD49d stimulation for 2 days.

Supplementary Figure 2 Plasma cytokines measurements. Plasma cytokines and markers of inflammation, including **(A)** IFN-γ, **(B)** tumor necrosis factor (TNF)-α, **(C)** IL-2, **(D)** IL-4, **(E)** IL-13, and **(F)** IL-17A, were ran in duplicates using a multiple immunoassay panel (MesoScale, Rockville, MD).
