# Supplementary figures and images for: Leptin Promotes Greater Ki67 Expression in CD4+ T Cells From Obese Compared to Lean Persons Living With HIV
Source: Front Immunol. 2022 Jan 17;12:796898. doi: 10.3389/fimmu.2021.796898 (PMC8801429; doi:10.3389/fimmu.2021.796898)

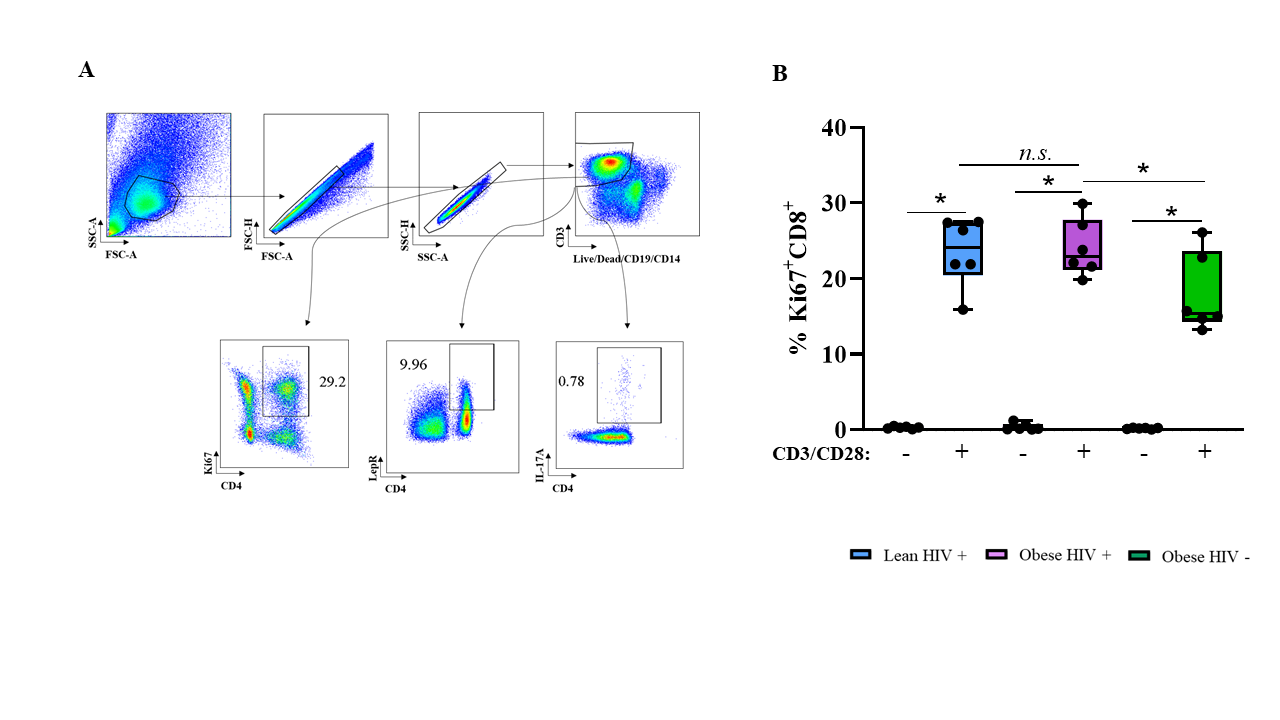

Supplement: Supplementary Figure 1 — Gating Strategy. (A) Gating Scheme, following selection of singlet viable CD3+CD19-CD14- T cells, we identified Ki67+CD4+ T cells, leptin receptor (LepR)+CD4+ T cells or IL-17A+CD4+T cells. (B) Quantification of the percentage of Ki67+CD8+T cells after anti-CD3/CD28/CD49d stimulation for 2 days. [file Image_1.tif]

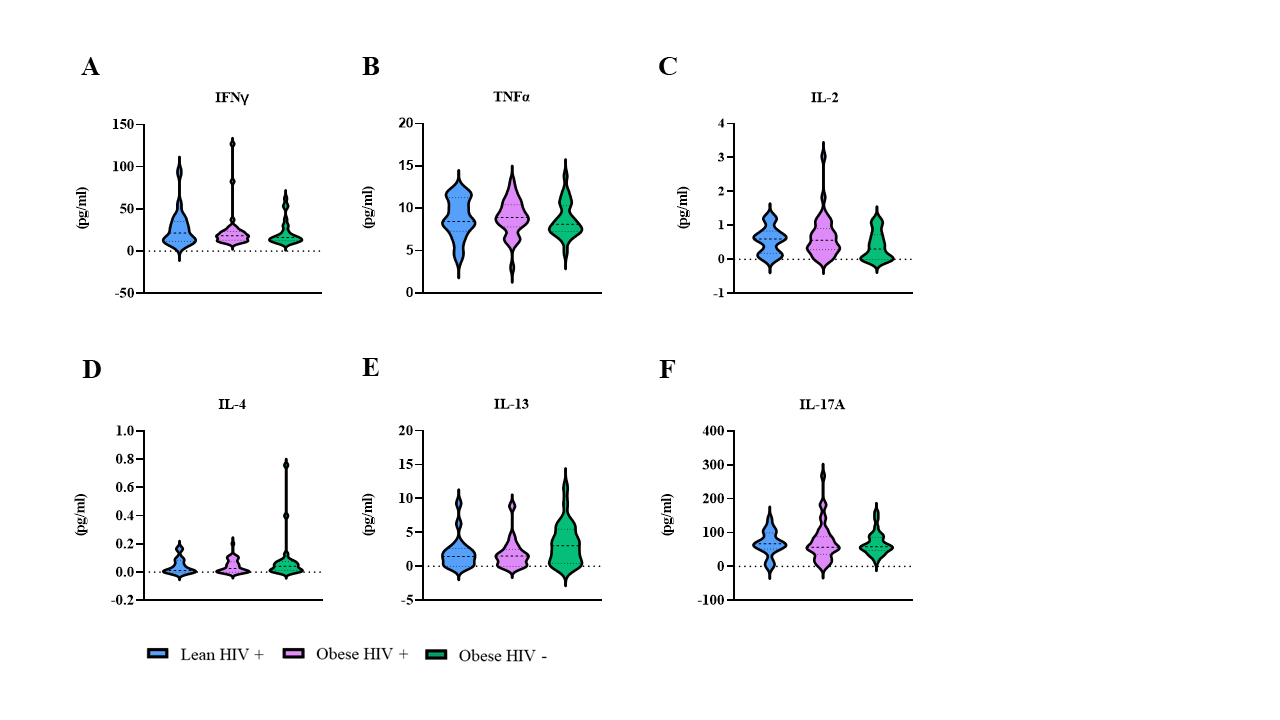

Supplement: Supplementary Figure 2 — Plasma cytokines measurements. Plasma cytokines and markers of inflammation, including (A) IFN-γ, (B) tumor necrosis factor (TNF)-α, (C) IL-2, (D) IL-4, (E) IL-13, and (F) IL-17A, were ran in duplicates using a multiple immunoassay panel (MesoScale, Rockville, MD). [file Image_2.tif]
